# Prognostic value of regulatory T cells and T helper 17 cells in high grade serous ovarian carcinoma

**DOI:** 10.1007/s00432-022-04101-2

**Published:** 2022-06-28

**Authors:** Sofya Marchenko, Iris Piwonski, Inga Hoffmann, Bruno Valentin Sinn, Catarina Alisa Kunze, Nanna Monjé, Jonathan Pohl, Hagen Kulbe, Wolfgang Daniel Schmitt, Sylvia Darb-Esfahani, Elena Ioana Braicu, Ann-Christin von Brünneck, Jalid Sehouli, Carsten Denkert, David Horst, Korinna Jöhrens, Eliane Tabea Taube

**Affiliations:** 1 Institute of Pathology, Charité – Universitätsmedizin Berlin, corporate member of Freie Universität Berlin and Humboldt Universität Zu Berlin, Berlin, Germany; 2 Tumorbank Ovarian Cancer Network, Berlin Institute of Health, Charité - Universitätsmedizin Berlin, corporate member of Freie Universität Berlin, Humboldt-Universität Zu Berlin, Berlin, Germany; 3 Department of Gynecology, European Competence Center for Ovarian Cancer, Charité Universitätsmedizin Berlin, corporate member of Freie Universität Berlin, Humboldt-Universität Zu Berlin, Berlin, Germany; 4 Institute of Pathology, Philipps-University Marburg, Marburg, Germany; 5 Institute of Pathology, Universitätsklinikum Carl Gustav Carus, TU Dresden, Dresden, Germany; 6 Institute of Pathology Berlin-Spandau, Berlin-Buch, Berlin, Germany

**Keywords:** Regulatory T-cells, High grade ovarian carcinoma, Prognosis, Tumor-infiltrating lymphocytes, Tumor microenvironment, Digital pathology

## Abstract

**Purpose:**

In recent years the tumor microenvironment and its interaction with the tumor has emerged into research focus with increased attention to the composition of Tumor-infiltrating lymphocytes. We wanted to quantify the composition of Regulatory T cells (Tregs) and T helper 17 cells (Th17 cells) and their prognostic impact in high-grade serous tubo-ovarian carcinoma.

**Methods:**

Tregs and Th17 cells were determined by immunohistochemical analysis of CD25 FoxP3 and RORγt, respectively on tissue microarrays of a cohort of 222 patients with reviewed histology and available clinical data. Expression was analyzed with Qupath for quantification and integration with clinical data enabled calculation of prognostic impact. For validation FOXP3 and RORC mRNA expression levels from 502 patients with HGSC in publicly available datasets were evaluated.

**Results:**

An average percentage of 0.93 Tregs and of 0.06 Th17 cells was detected per cells in overall tissue. Optimal cut-offs were determined and higher Tregs were associated with a better overall survival in stroma (*p* = 0.006), tumor area (*p* = 0.0012) and overall tissue (*p* = 0.02). After accounting for well-known prognostic factors age at diagnosis, residual tumor and FIGO stage, this association remained significant for stromal Tregs with overall survival (*p* = 0.02). Survival analysis for Th17 cells revealed no significant association with survival rates. Moreover, lower Th17/Treg ratios had a positive impact on patient overall survival (*p* = 0.025 tumor, *p* = 0.049 stroma and *p* = 0.016 overall tissue).

**Conclusion:**

Our results outline a positive prognostic effect for higher Tregs but not for Th17 in high grade serous tubo-ovarian carcinoma.

**Supplementary Information:**

The online version contains supplementary material available at 10.1007/s00432-022-04101-2.

## Introduction

Ovarian cancer is the 8th most frequently diagnosed cancer type for women in 2020, accounting to 3.4% of the total number of cancer cases and it has led to approximately 207,252 new deaths in 2020 worldwide (Sung et al. 2021). With a mortality rate of 6.7 per 100,000 women in 2018, ovarian cancer is the most common cause of death among gynecological malignancies. High-grade serous ovarian carcinoma (HGSC) is the most prevalent histology subtype of epithelial ovarian cancer (EOC) which is diagnosed in 70% of all cases (Reid et al. 2017). Since the early stage of this disease is typically asymptomatic, it is often not diagnosed until late stages (FIGO III/IV), which account for poor patient prognosis, with only half of the patients surviving for more than five years after being diagnosed (Torre et al. 2018).

The standard treatment for advanced-stage HGSC—debulking surgery together with a platinum-based chemotherapy—comes at several limitations, such as drug resistance with subsequent recurrence in 60–70% of all cases (Ozols et al. 2003; Wang et al. 2017). Moreover, it is commonly known that this treatment is associated with severe side effects (Wang et al. 2017). Hence, optimization of medication and reduction of side effects through prolongation of recurrence free intervals and personalized therapy are needed. Apart from cancer cells, the cells of the tumoral microenvironment, especially the tumoral immune cells, can serve as a potential prognostic or therapeutic marker. The intra- and peritumoral immune cells are a heterogenous group of several different cell types (Sun and Zhang 2014). To this end, tumor infiltrating lymphocytes (TILs) are becoming a source of growing interest. Ongoing research has demonstrated that the composition of TILs in tumors is unique, and proportions of certain immune cells can serve as reliable predictors of the patient’s survival. Furthermore, immune cells have the potential to sustain oncologic treatment.

The discovery that programmed cell death protein 1 (PD-1) and cytotoxic T-lymphocyte-associated protein 4 (CTLA-4) expressed on the surface of T cells act as a “brake” in immune function led to an approach to target cancer. A treatment has been developed by immune checkpoint inhibition that reactivates T cells and eliminates cancer cells more effectively (Nobel Prize Outreach 2021). Due to these findings, cancer research had a strong focus on T-Helper cells in the past years. Early research focused especially on relation of Th1- and Th2-helper cells. After the discovery of the additional subgroups, the Th17 and the regulatory T cells (Tregs), a lot of additional information surfaced on this topic (Duan et al. 2014). Th1, Th2, Th17 and Tregs interact in multiple complex and cross-linked pathways among each other and the whole TME. Especially Tregs and Th17 cells can impair or support in direct or more often in indirect ways the effect of Th1 and Th2 (Govindaraj et al. 2013; Chaudhary and Elkord 2016).

In clinical setting, a meta-analysis evaluating 76 studies revealed that Treg prognostic value is highly dependent on tumor type and tumor stage (Shang et al. 2015). For ovarian cancer findings regarding the role and clinical value remain controversial. On one hand, Tregs have been demonstrated to play a crucial role in ovarian cancer pathogenesis by suppressing endogenous tumor-associated antigen specific T cell immunity (Curiel et al. 2004). Moreover, accumulation of Tregs in tumor tissue is known to have an enhanced effect on tumor progression and hence Tregs pose a major challenge for immunotherapies (Arce Vargas et al. 2017). Furthermore, a high ratio of effector T cells (Teff) to Treg cells, was shown to indicate improved overall survival of multiple cancer types (Shang et al. 2015). On the other hand, studies have found the opposite effect, namely, that Tregs are a favorable prognostic factor (e.g. (Leffers et al. 2009; Milne et al. 2009)). Tregs and activated T cells are characterized by the expression of the alpha subunit of the transmembrane receptor for Inter Leucin-2 (IL-2) on their cell surface (Minami et al. 1993) and by the transcription factor Forkhead Box P3 (FOXP3) (Hori et al. 2003).

Th17 cells, long perceived as the opponent of Tregs, are proinflammatory cells and produce the cytokine interleukin 17 (IL-17). The differentiation of naïve CD4 + T helper into Th17 is controlled by RORγt, a splicing isoform of RORγ, which has been demonstrated in an in vitro study (Ivanov et al. 2006). Retinoic acid receptor-related orphan receptors (RORs) are a superfamily of nuclear transcription factors that are known to co-regulate multiple (patho-) physiological processes including the circadian rhythm, reproductive development and metabolic disorders (Solt and Burris 2012).

As Th17 cells are of pro-inflammatory nature whereas Tregs act in an anti-inflammatory way and studies reporting their heterogeneous occurrence across different tumor entities, the aim of this study was to elucidate the clinical relevance of these TILs for HGSC. Detailed insights into the prognostic and diagnostic value of Tregs and Th17 cells and their interaction or else ratio will provide the basis for further research that eventually can contribute to the identification of potential therapeutic targets for patients with HGSC. Therefore, we aimed to determine the presence of Tregs and Th17 in the tissues of our HGSC study cohort. Moreover, we were interested whether the proportions of Tregs and Th17 cells can predict survival rates of patients with HGSC and hence serve as robust prognostic markers. For this, we evaluated immunohistochemistry stainings (IHC) of a large cohort of patients with HGSC with reviewed histology and available clinical data. For validation of the IHC results bulk mRNA expression levels from publicly available data sets of HGSC patients were analyzed.

## Materials and methods

### Study population

For the study, we included a total of 222 patients with high grade serous ovarian cancer from the Institute of Pathology of the Charité University Hospital who underwent cytoreductive surgery in the period from 1991 until 2009. Cases were identified from a local pathology archive (Nexus) in cooperation with the tumor bank ovarian cancer project (http://www.toc-network.de). Pathology review was performed on all cases by two board approved pathologists specialized in gynecologic pathology (S.D.E., E.T.T,) according to WHO criteria from 2014 (Kurman et al. 2014). The patient’s ages ranged from 34 to 87.13 years (median = 60.14 years). Cases with poor staining quality or missing clinical data were excluded. Cases containing only tumoral or only stromal tissue in the cores cause differences in the number of evaluable patients across the experiments (Table 1, for detailed numbers see S1). Classification of HGSC was done according to the criteria from International Federation of Gynecology and Obstetrics (FIGO). The conduction of this study has been approved by the local ethics committee (EA1/051/18) and supported by the TRANSCAN-2 project (grant no.:2014–121).

**Table 1:** Clinical characteristics of the study cohort

Parameter	Tregs OS		Tregs PFS		Th17 OS		Th17 PFS	
Tumor tissue	Cases	Percentage	Cases	Percentage	Cases	Percentage	Cases	Percentage
Total number of cases	198		198		199		199	
Number of evaluable cases	193	100.0	178	100.0	191	100.0	173	100.0
Age (years) <= 60	99	51.3	94	52.8	97	50.8	89	51.4
Age (years) >= 60	94	48.7	84	47.2	94	49.2	84	48.6
FIGO stage I–II	24	12.4	22	12.4	20	10.5	18	10.4
FIGO stage III–IV	169	87.6	156	87.6	171	89.5	155	89.6
postoperative residual tumor	49	25.4	44	24.7	52	27.2	46	26.6
no residual tumor	91	47.2	89	50.0	91	47.6	87	50.3
no data for R status	53	27.4	45	25.3	48	25.1	40	23.1
Stroma tissue								
Total number of cases	176		176		200		200	
Number of evaluable cases	172	100.0	156	100.0	192	100.0	173	100.0
Age (years) <= 60	87	50.6	81	51.9	96	50.0	88	50.8
Age (years) >= 60	85	49.4	75	48.1	96	50.0	85	49.1
FIGO stage I–II	23	13.4	21	13.5	21	10.9	19	11.0
FIGO stage III–IV	149	86.6	135	86.5	171	89.1	154	89.0
postoperative residual tumor	43	25.0	38	24.4	52	27.1	46	26.6
no residual tumor	79	45.9	76	48.7	90	46.9	85	49.1
no data for R status	50	29.1	42	26.9	50	26.0	42	24.3
Overall tissue								
Total number of cases	196		196		204		204	
Number of evaluable cases	191	100.0	177	100.0	196	100.0	177	100.0
Age (years) <= 60	98	51.3	93	52.5	99	50.5	91	51.4
Age (years) >= 60	93	48.7	84	47.5	97	49.5	86	48.6
FIGO stage I–II	24	12.6	22	12.4	21	10.7	19	10.7
FIGO stage III–IV	167	87.4	155	87.6	175	89.3	158	89.3
postoperative residual tumor	49	25.7	44	24.9	52	26.5	46	26.0
No residual tumor	90	47.1	88	49.7	94	48.0	89	50.3
No data for R status	52	27.2	45	25.4	50	25.5	42	23.7

### Immunohistochemistry

Formalin-fixed and paraffin-embedded (FFPE) surgical specimens of the patients were used to prepare TMAs with two cores per patient of 1.0 to 1.5 mm diameter for immunohistochemistry (IHC). IHC was performed using a Leica Bond Master, according to the manufacturer’s instructions. Heat-induced antigen retrieval was performed with EDTA buffer. For the detection of Tregs a double staining with fast red labeled antibodies against CD25 (clone 4C9, solution 1:50, Leica Biosystems, Buffalo Grove, USA) and FOXP3 (clone 236A/E7, solution 1:200, Abcam plc., Cambridge, UK) was performed. We used a double staining, to target exclusively Tregs, as it is known that FOXP3 can be expressed on tumor or epithelial cells as well. For detection of Th17 cells the rabbit polyclonal Anti-RORγt labeled by Diaminobenzidine (DAB) (solution 1:100, Abcam plc., Cambridge, UK) was used.

### Immunohistochemical quantification

To quantify the frequencies of Tregs and Th17 cells, respectively, the open-source Software QuPath (v0.2.0-m10) was used for bioimage analysis (Bankhead et al. 2017). First, tumoral and stromal compartments were labeled manually in each TMA core by a trained person (S.M.). The labelling process was controlled by a trained pathologist (I.P.) for the first 50 cases and for every case with doubts. The labelling process was kept continuous for all samples. For internal quality assurance, we evaluated the correlation strength of tumor-to-stroma ratios (TSR), as a similar ratio indicates the stromal and tumoral area are equally distributed between the two markers in the same TMA core and found good accordance (see 3.1). Finally, positive cells in the TMA cores were detected by an inbuilt QuPath algorithm ("positive cell detection") for the overall core and for stroma and tumor tissue separately. To select parameters for nucleus size and variation as well as intensity thresholds visual inspection was done by a trained pathologist (E.T.T.) on a subset of 10 TMA cores across five different TMAs. In this process of finding the parameters, manual annotations reinforced the choice of parameters, as QuPath uses semi-supervised machine learning. A detailed list of the final applied parameters can be found in the supplementary material (S2). Afterwards, all TMA cores were manually inspected, and major artifacts were excluded by a trained person (S.M). Finally, validation of automated IHC cell quantification was performed by two experienced pathologists (E.T.T, I.P.) on areas of size 250 × 250 µm from 30 randomly chosen TMA cores per staining. To assess the agreement between the automated cell count and manual scoring, intraclass correlation coefficient (ICC) was computed for total and positive cell count, respectively. Afterwards, to normalize the data for the total area of tumor and stroma, positive cell counts were divided by total cell counts yielding intuitive percentage values. For downstream analysis, we selected one TMA core per patient – the core with higher tumor tissue content. Due to staining quality issues, a minority of samples were excluded from downstream analysis (see S1). Taken together with missing clinical data, the total case number resulted in 193 annotated cores for Tregs and 204 patients for Th17 with an intersection of *n* = 177 patients with available percentage values for both Th17 and Tregs.

### Statistical analysis

Statistical analysis was performed using the statistical software SPSS (IBM SPSS Statistics Version 27.0.0.0 64-Bit-Version) and R (version 4.0.4). The following procedure was repeated three times for each marker: for tumor and stroma values and for overall tissue (values for tumor and stroma combined), respectively. Cutoff values were determined using the web application “Cutoff Finder” from the University Heidelberg and, patients were stratified into groups with high and low proportion of positive cells (Budczies et al. 2012). The optimal cutoff was defined as the point with the most significant (log-rank test) split. To reduce type 2 errors, significance was only assessed if at least 5% of the tested cut-offs yielded statistically significant difference in overall survival. For a comparison of the grouped data with the respective optimal cutoffs, we performed survival analyses with Kaplan–Meier plots. The log-rank test was used to compare the survival curves in overall survival (OS) as well as progression-free survival (PFS). PFS was defined in patients with follow-up care as the time from cancer diagnosis to first recurrence of disease measured by clinical symptoms and medical imaging, as in Germany rebiopsy in recurrent situation is not performed routinely. Patients without a defined event (either “alive” or “death during follow-up” or not in follow-up care for PFS at the time of the analysis) were censored in statistical analysis at the time of last follow-up. Moreover, to account for the known confounding factors, we performed multivariate analysis (MVA), with the following confounders considered: age at diagnosis, the presence of residual tumor and FIGO stage. Analogously, the analysis steps were performed for Th17/Treg ratios on absolute cell count ratios. To investigate the proportions of positive cells according to FIGO stages, boxplots were computed, and the nonparametric Mann–Whitney *U* test was applied to observe whether there are any detectable significant differences (FIGO I/II vs. FIGO III/IV). As the study design was explorative, no correction for multiple testing was performed. Correlation strength between tumor-to-stroma ratios was assessed using the Spearman’s rank correlation coefficient. P-values below 0.05 were considered statistically significant.

### In silico validation on gene expression level

Differences in bulk mRNA of genes encoding the proteins FOXP3 and RORγt were investigated on a separate cohort to compare our histochemical results on mRNA level. Cut-offs and survival curves were computed for the genes *FOXP3* (Affymetrix-ID: “224211_at”) to validate the impact of Tregs and *RORC* (Affymetrix-ID “206419_at”) for Th17 cells, respectively. To generate survival curves, we used the online tool Kaplan–Meier plotter (Gyorffy et al. 2012). We selected only serous carcinoma of either grade 2 or grade 3 and used OS as the outcome. We used all available probe sets per gene and excluded outlier arrays. This procedure led to a total sample size of 502 patients for both RORC and FOXP3 respectively. Furthermore, we investigated whether the ratio of the genes RORC and FOXP3 has an impact on the overall survival of patients. The data used, originated from multiple publicly available data sets: GSE18520 (*n* = 20) (Mok et al. 2009), GSE27651 (*n* = 11) (King et al. 2011), GSE30161 (*n* = 43) (Ferriss et al. 2012), GSE23554 (*n* = 26) (Marchion et al. 2011), GSE63885 (*n* = 57) (Lisowska et al. 2016), GSE9891 (*n* = 261) (Tothill et al. 2008), and GSE26193 (*n* = 100) (Mateescu et al. 2011). Data used in the Kaplan–Meier Plotter stems only from publications with available raw microarray gene expression data and clinical survival information. Gene expression data included in the KM plotter were measured by only three microarray platforms, GPL96 (Affymetrix HG-U133A), GPL570 (Affymetrix HG-U133 Plus 2.0), and GPL571/GPL3921 (Affymetrix HG-U133A 2.0), which are frequently used and all three have 22, 277 probe sets (representing 13, 435 unique genes) in common. The raw. CEL files were MAS5 normalized using the affy R/Bioconductor library (Torre et al. 2018). Moreover, the mRNA data underwent a second scaling normalization which sets average expression on each chip to 1000. This technique significantly reduces batch effects (Ozols et al. 2003).

## Results

### Expression of Tregs and Th17

**Fig. 1:**
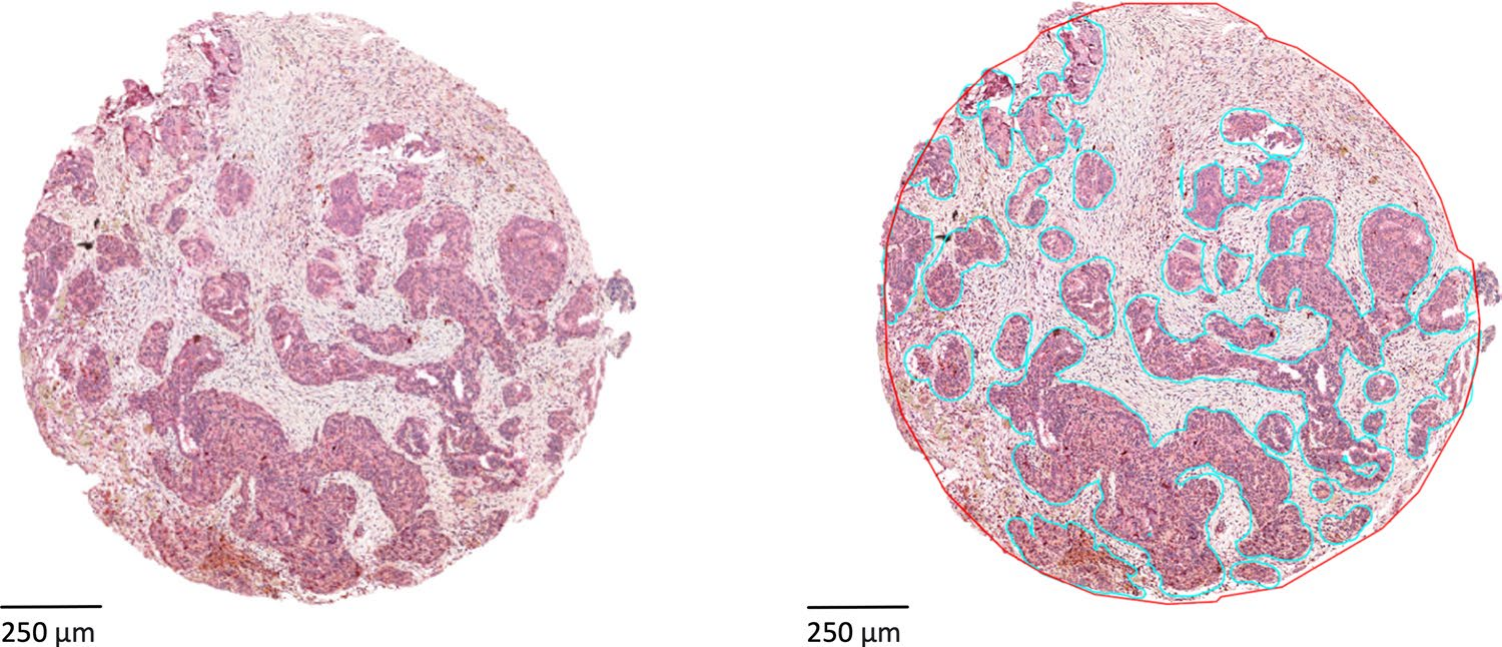
QuPath processing results. **A** Representative CD25 FOXP3 stained HGSC TMA core. **B** The corresponding TMA core after applying automated simple tissue detection (outer red line) and manually annotated tumor area marked by cyan line

**Fig. 2:**
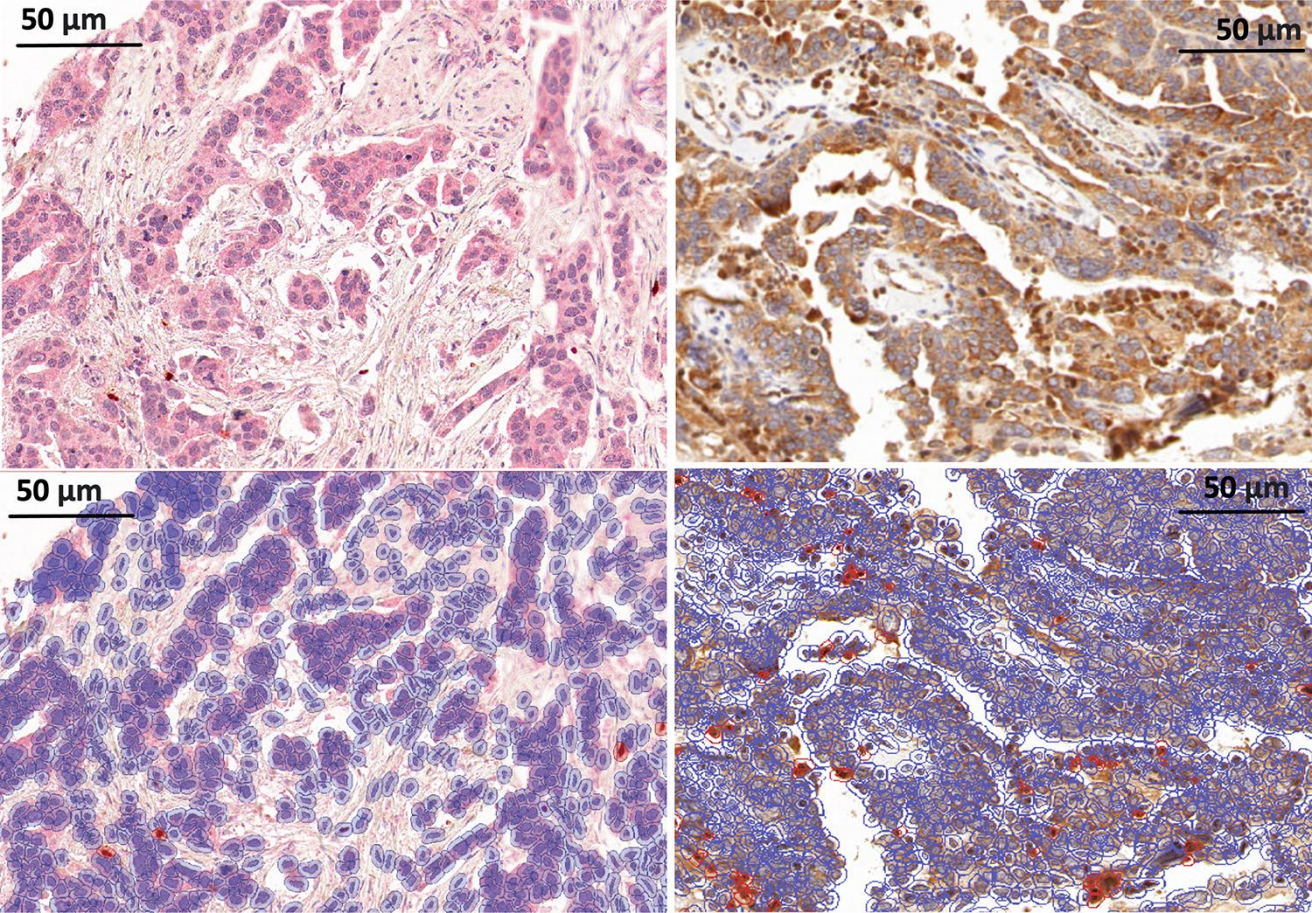
HGSC Staining and positive cell detection results. **A** Representative CD25 FOXP3 stain. **B** Same area as shown in A after positive cell detection, with detected cells highlighted in blue and red highlighted CD25 + FOXP3 + positive cells. **C** Representative RORγt staining result. **D** same core as in C after positive cell detection

**Fig. 3:**
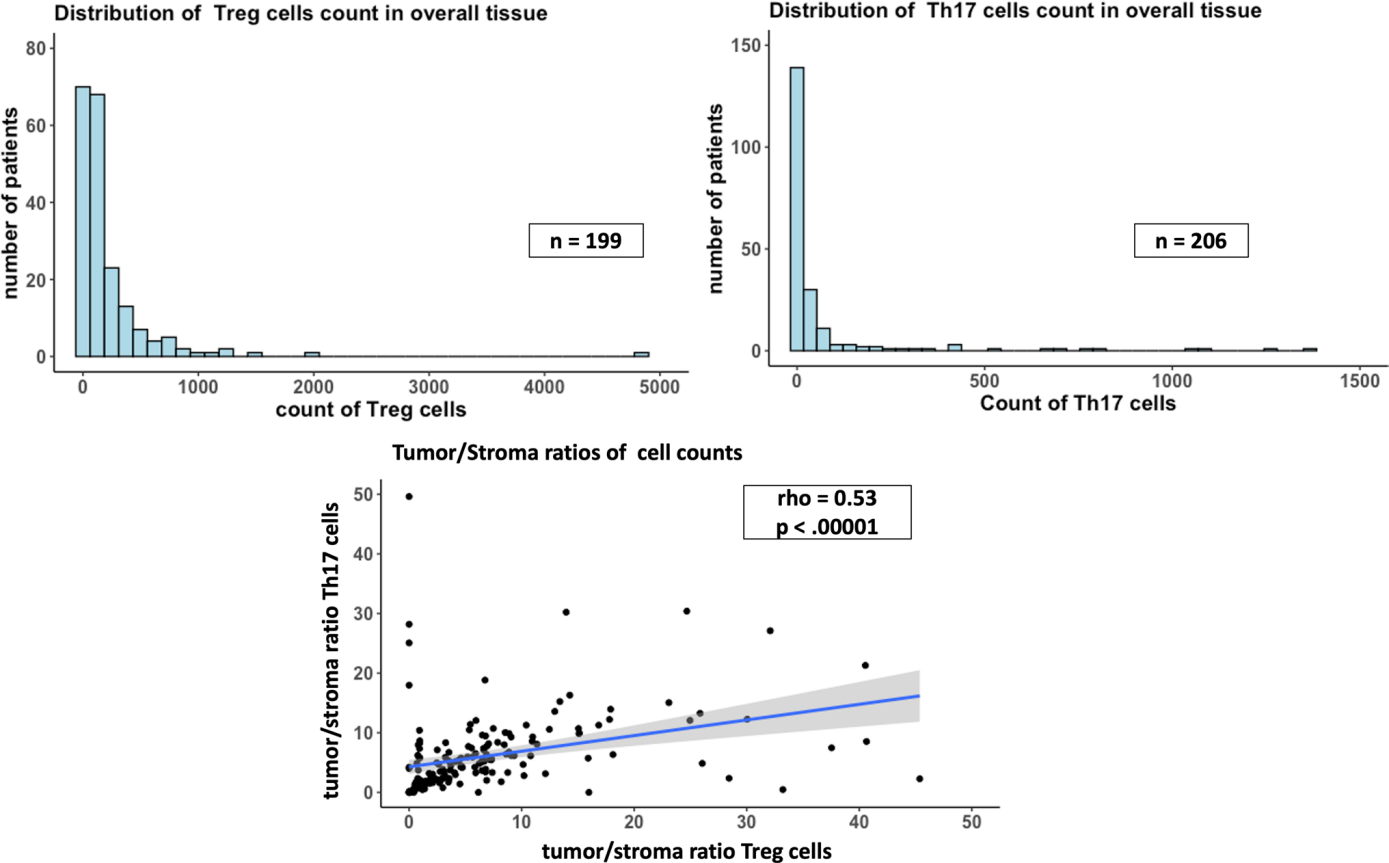
Quantitative results from staining and manual tissue detection**.** **A** Distribution showing amounts of patients per count of detected Treg cells. **B** Distribution showing amounts of patients per count of detected Th17 cells. **C** Correlation of tumor/stroma ratios of total cell counts between Th17 and Tregs

For the TMA analysis, a typical QuPath Workflow has been applied, which included several pre-processing steps on the TMAs. The results from the key steps that are crucial for downstream analysis are presented here. A representative TMA core and the results of the QuPath tissue detection and compartment annotation are illustrated in Fig. 1. On the annotated TMA cores, we applied positive cell detection (displayed on examples for each staining in Fig. 2), which revealed that Treg cells were detected in most of the samples, whereas Th17 cells were less prevalent. For both cell types we observed a left skewed distribution (Fig. 3A, B). The mean percentage of Treg cells per TMA core was 0.93, while the average cell percentage of Th17 was only 0.06 positive cells per core. Moreover, for Th17 we found 16 of 206 TMA cores (7.8%) with no positive cells detectable, while for Tregs this was only true for one out of 174 TMA cores (0.5%). Validation of the automated cell count revealed an ICC of 0.81 (first board approved pathologist) and 0.68 (second board approved pathologist) for the total cell count in Tregs and an ICC of 0.89 and 0.75 for the total cell count in Th17. Here, similar ICCs were found between the observers (0.89 for Treg analysis and 0.84 for Th17 analysis). Likewise, for positive cell count, also a strong agreement between board approved pathologists and automated cell detection was found (see S3). Regarding the tumor-to-stroma ratios (TSR) for absolute cell count, the results were in alignment with our previous expectations: a positive correlation with some heavy outliers (Spearman rho 0.53, *p* < 0.00001). As depicted in Fig. 2C, smaller TSRs show a stronger correlation and higher ratios (more tumor tissue than stroma tissue in a TMA core) tend to deviate more from the diagonal line, which represents a perfect correlation. The scattering of the TSR between the two markers can be explained by the fact that the tissues were stained at different cutting depths. Moreover, the manual annotation together with cell detection also contributes to minor errors.

### Cell frequencies in different tumor stages

**Fig. 4:**
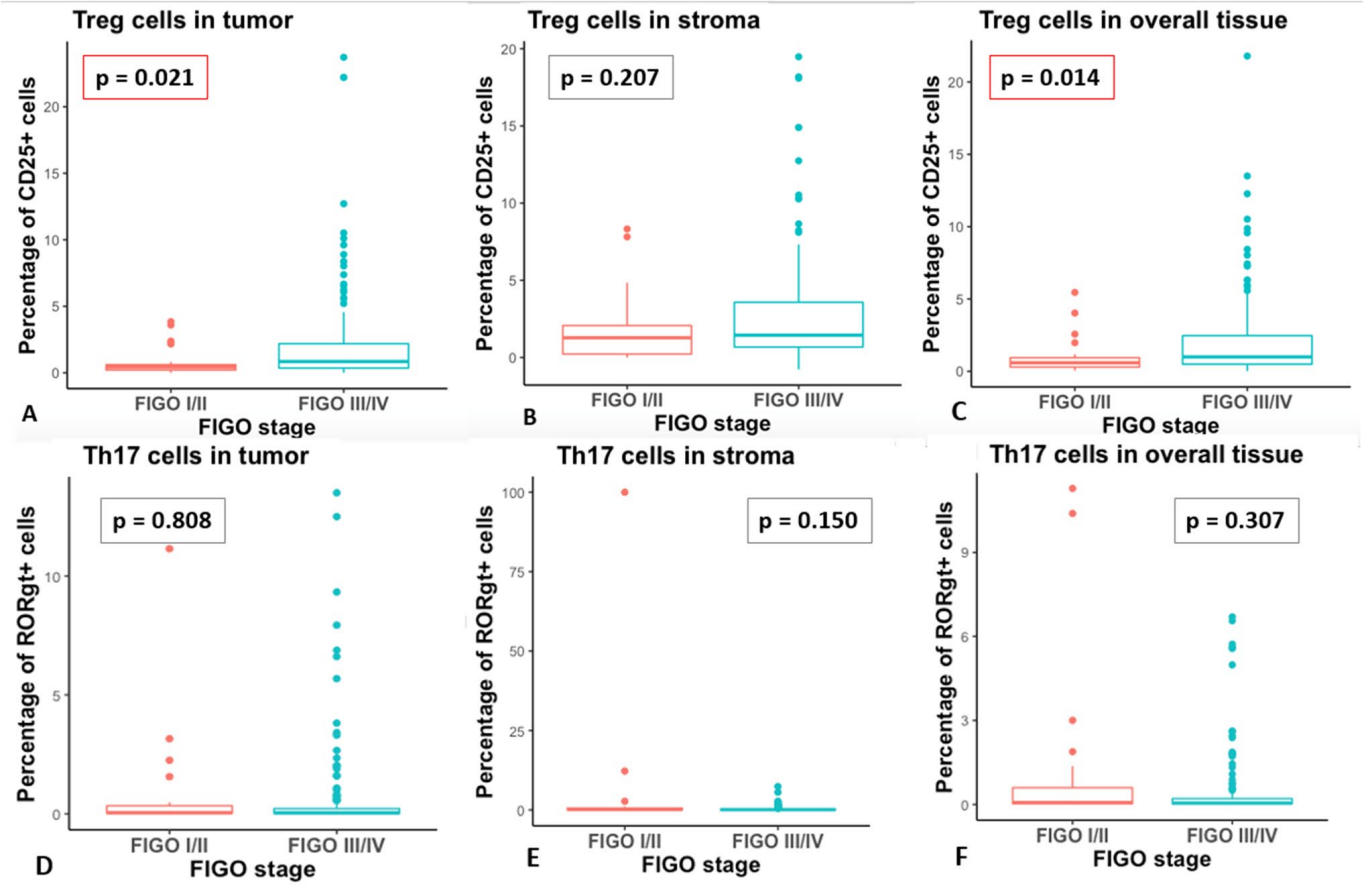
Boxplots comparing the frequency of positive cells depending on patient’s FIGO status **A** In tumor Treg frequencies are significantly higher in advanced stages. **B** In stroma there is a trend towards higher Treg amounts in advanced FIGO stages. **C** In overall tissue Treg frequencies are significantly higher at advanced stages. **D**–**F** Th17 frequencies show no association with the FIGO stages independent of the tissue area that was investigated

Subsequently, we examined how positive cell frequencies vary among the different tumor stages. Patients were grouped by their FIGO stages: the early stages FIGO I/II on one hand vs. the advanced stages FIGO III/IV on the other hand. As shown in Table 1, the majority of the patients included in the study had an advanced FIGO stage (FIGO III or IV). For Tregs, the frequencies of positive cells in tumor (*p *= 0.021, effect size *r* = 0.17, Mann–Whitney U-test) and overall tissue (*p* = 0.014, effect size *r* = 0.18, Mann–Whitney U-test) showed significant differences even though with little effect sizes (Fig. 4A, C): the tissue from advanced stages showed an increased percentage of positive cells compared to patients at lower stages. In contrast, for Th17 frequencies and Treg frequency in stromal tissue no significant differences between patients’ FIGO status were observed (Fig. 4B, D, E, F).

### Prognostic impact of Tregs and Th17 cells

**Fig. 5:**
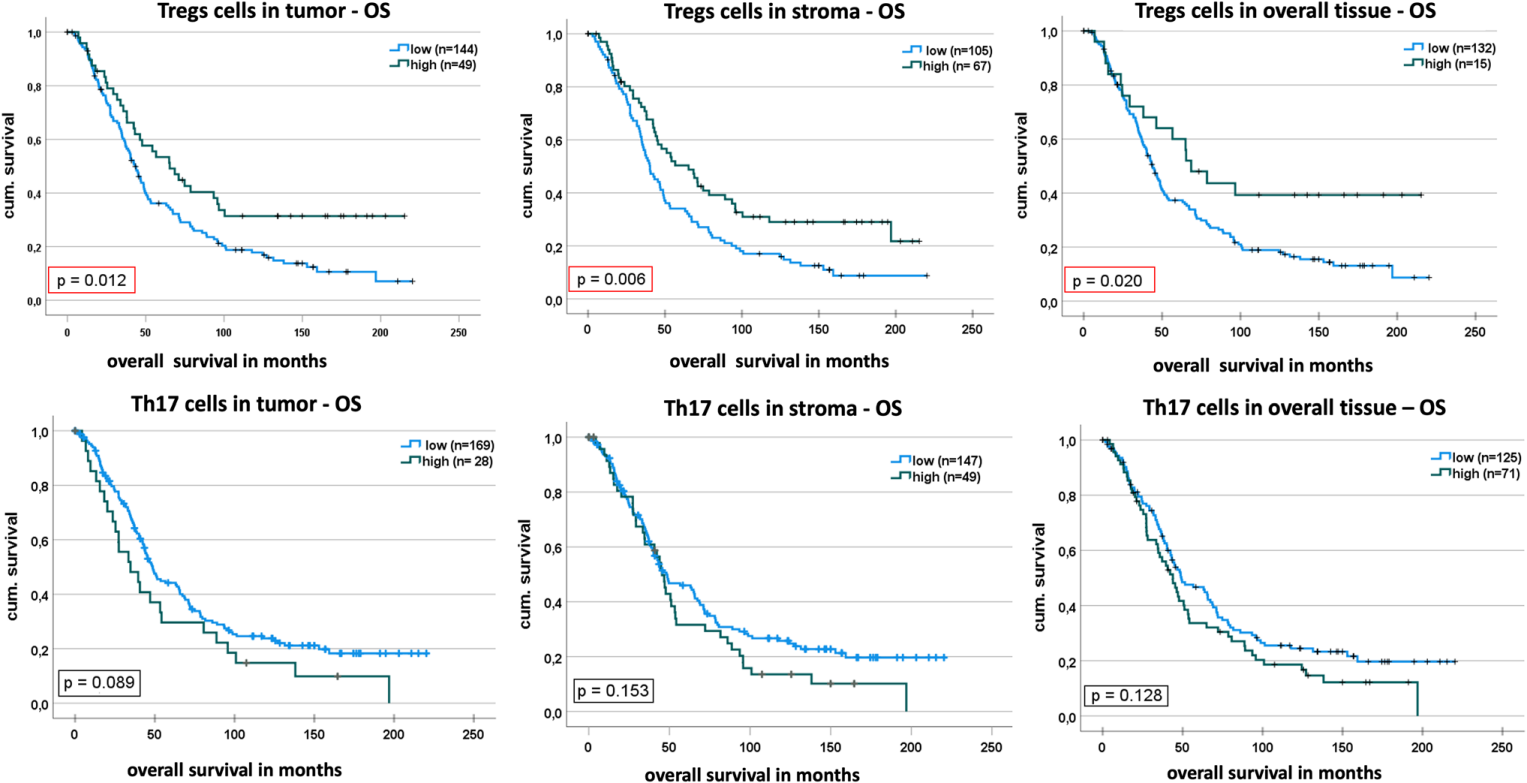
Kaplan–Meier survival curve showing overall survival depending on positive cell frequency divided by optimal cut-off into two groups**.** **A** Overall survival in patients with high Treg frequencies in tumor is significantly longer. **B** Higher Treg frequencies in stroma have as well a significant impact on the OS. **C** OS increases with higher Treg content of the overall tissue. **D** No association between OS and Th17 frequencies in tumor. **E** Same holds true for OS and Th17 cell frequencies in stromal tissue. **F** OS depending on Th17 frequencies in overall tissue did not reveal a significant difference between the groups

As positive cell detection and tissue annotation led to satisfying results, we preceded by assessing the impact of the relative cell counts on overall and progression free survival of the patients in the study cohort. This was done for Tregs and Th17 cells in tumor, stroma and overall area. The cut-offs were determined as described in material and methods, for detailed information and overview of our statistical results see Table 2. The median overall survival for the patient group for which Tregs were found, ranged from 45.4 to 46.3 months (different median values are due to patient group sizes varying slightly depending on the occurrence of tumoral and stromal area in the considered core). Increased Treg frequency was strongly associated with a prolonged overall survival irrespective of the investigated area in the core (Fig. 5A, B, C). The significance was highest for OS in stroma (*p* = 0.006), followed by tumor area (*p* = 0.0012) and overall tissue (*p* = 0.02). For stroma tissue evaluation, the median OS in the group of high Tregs was 65 months (95%CI 43.1–87.5) as opposed to 40 months (95% CI 32.9–47.7) for the low Tregs group. For tumor tissue evaluation, the median OS in the group of high Tregs was also 65 months (95% CI 43.1–87.5 months) as opposed to 43 months (95% CI 37.5–48.9 months) for the low Tregs group. For tumor and stromal areas taken together, the median OS was 69 months in the high Treg group, while the low Treg group had a median OS of 44 months (95% CI 38.3–49.5). After accounting for well-known prognostic factors age at diagnosis, residual tumor and FIGO stage, only Treg frequency in stroma remained significantly associated with OS (*p* = 0.02). Even though considerably less, Tregs were still associated with a longer PFS for tumor tissue even after accounting for confounders (UA: *p* = 0.031, MVA: *p* = 0.034). In this analysis, patients with high Treg frequencies in tumor had a median PFS of 29 months (95% CI 21.7–36.0) while the patients grouped below the cut-off value had a median PFS of 18 months (95% CI 14.2–21.9). In stromal area we did not observe any association between Treg frequency and PFS. Moreover, there exist a range of cut-offs that yield a significant association between OS and Treg frequency, which indicates that Tregs is a robust marker (Figure S4). For Th17 frequencies we found no statistically significant results (Fig. 5D, E, F). For these cases neither tumor tissue (*p* = 0.089), nor overall tissue (*p* = 0.128) and stroma area (*p* = 0.153) showed statistically significant differences in survival rates. Also, for PFS we could not detect any significant association with Th17.

**Table 2:** Results from statistical survival analysis including optimal cut-offs

Frequency/Ratio	*n* patients	median survival in months (95% CI)	Cut-off	Entire Cohort Analysis						FIGO III/IV Analysis		
				*n* patients <= cut-off	*n* patients > cut-off	median surival in months (95% CI)-<=cut-off (low)	median surival in months (95%CI) > cut-off (high)	UA^1^ *p*-value	MVA^2^ *p*-value	cut-off	UA^1^ *p*-value	MVA^2^ *p*-value
Tregs –OS												
Tumor	193	46.3 (41.3–51.4)	2.115	144	49	43.2 (37.5–48.9)	65.3 (42.8–87.8)	0.012*	0.171	2.19	0.012*	0.168
Stroma	172	45.4 (38.8–51.9)	2.068	105	67	40.3 (32.9–47.7)	65.3 (43.1–87.5)	0.006*	0.020*	2.09	0.006*	0.027
Overall	191	46.3 (41.3–51.35)	3.905	166	25	43.9 (38.3–49.5)	68.5 (46.8–90.2)	0.020*	0.194	3.905	0.028*	0.216
Tregs –PFS												
Tumor	178	20.5 (16.6–24.5)	2.01	131	47	18.1 (14.2–21.9)	28.9 (21.7–36.0)	0.03*	0.034*	43497	0.016*	0.019*
Stroma	156	21.6 (16.8–26.5)	6.125	96	60	20.5 (16.0–25.0)	39.2 (13.1–65.3)	0.03*	0.196	3565	0.022*	0.062
Overall	177	20.7 (16.6–24.8)	3.905	153	24	18.9 (15.8–22.0)	29.3 (13.9–44.6)	0.04*	0.096	3905	0.024*	0.062
Th17 –OS												
Tumor	197	47.05(41.4–52.7)	0.5172	169	28	48.7 (41.3–56.2)	34.7 (14.1–55.3)	0.089	0.484	0.255	0.121	0.202
Stroma	196	46.75(41.0–52.5)	0.2128	147	49	48.1 (31.7–64.7)	45.3 (39.4–51.3)	0.153	0.591	0.785	0.272	0.247
Overall	196	47.05(41.1-52.7)	0.105	125	71	49.2 (32.6–65.8)	43.9 (33.8–53.9)	0.128	0.245	0.105	0.188	0.254
Th17.–PFS												
Tumor	177	21.1 (16.4–25.8)	0.015	45	128	18.8 (10.8–26.9)	21.4 (15.5–27.3)	0.272	0.144	0.085	0.170	0.144
Stroma	173	22.3 (17.4–27.1)	0.035	64	109	24.7 (20.9–28.5)	20.7 (13.9–27.5)	0.14	0.728	1405	0.137	0.368
Overall	177	21.2 (16.3–26.1)	0.005	17	160	14.8 (6.2–23.3)	23.2 (17.4–29.0)	0.159	0.272	0.035	0.102	0.011*
Th17/Tregs –OS												
Tumor	166	46.8 (40.5–53.0)	1.17	135	31	49.2 (32.9–65.6)	34.7 (17.9–51.5)	0.02*	0.200	1.17	0.04*	0.231
Stroma	160	45.4 (40.1–50.7)	0.095	98	25	49.6 (29.8–69.5)	42.2 (32.7–51.7)	0.04*	0.268	0.025	0.196	0.557
Overall	170	45.4 (40.1–50.7)	0.715	141	29	48.9 (33.3–64.5)	33.5 (22.1–44.9)	0.01*	0.195	11689	0.022*	0.307
Th17/Tregs –PFS												
Tumor	153	21.2 (15.7–26.7)	3.05	135	18	23.2 (16.4–29.5)	16.4 (7.7–25.1)	0.242	0.67	2.125	0.233	0.605
Stroma	146	20.7 (15.6–25.8)	0.055	69	77	25.0 (18.0–32.1)	17.4 (11.9–22.9)	0.037*	0.311	0.055	0.048*	0.274
Overall	156	21.1 (16.1–26.1)	0.015	33	123	22.3 (12.5–32.0)	20.7 (14.5–27.0)	0.283	0.952	0.755	0.219	0.306

^1^Univariate analysis

^2^Multivariate analysis with known prognostic factors (age, FIGO stage, residual tumor

### Correlation of positive cell frequencies in Tregs and Th17

**Fig. 6:**
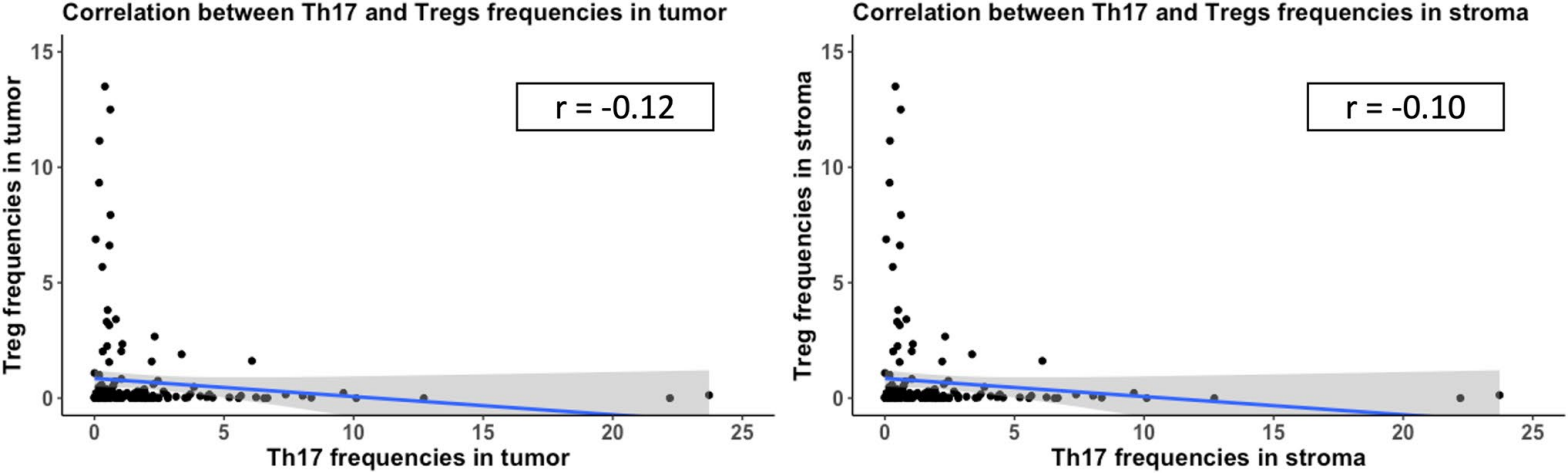
Correlation between Treg frequencies and Th17 together with spearman correlation coefficients (rho). **A** For tumor area **B** for positive cell percentages in stroma

As we observed that higher Treg percentages are significantly associated with a longer survival, while no statistical significance was observed for Th17 cells, we investigated the correlation between Treg cells and Th17 cells and indeed we observed a decent negative relationship, with patients having higher percentages of Tregs tend to have lower frequencies of Th17 cells. This slight trend is present in all three tissue types tumoral and stromal and overall, with similar correlation strengths (Pearson correlation coefficient varies between –0.1 for stroma (lowest) to –0.13 for overall tissue) (Fig. 6A, B). However, as the data is not normally distributed and the correlation strength is very weak, we did not go beyond plotting the relationship for exploratory purpose.

### The ratio of Treg and Th17 is associated with prolonged survival in HGSC

**Fig. 7:**
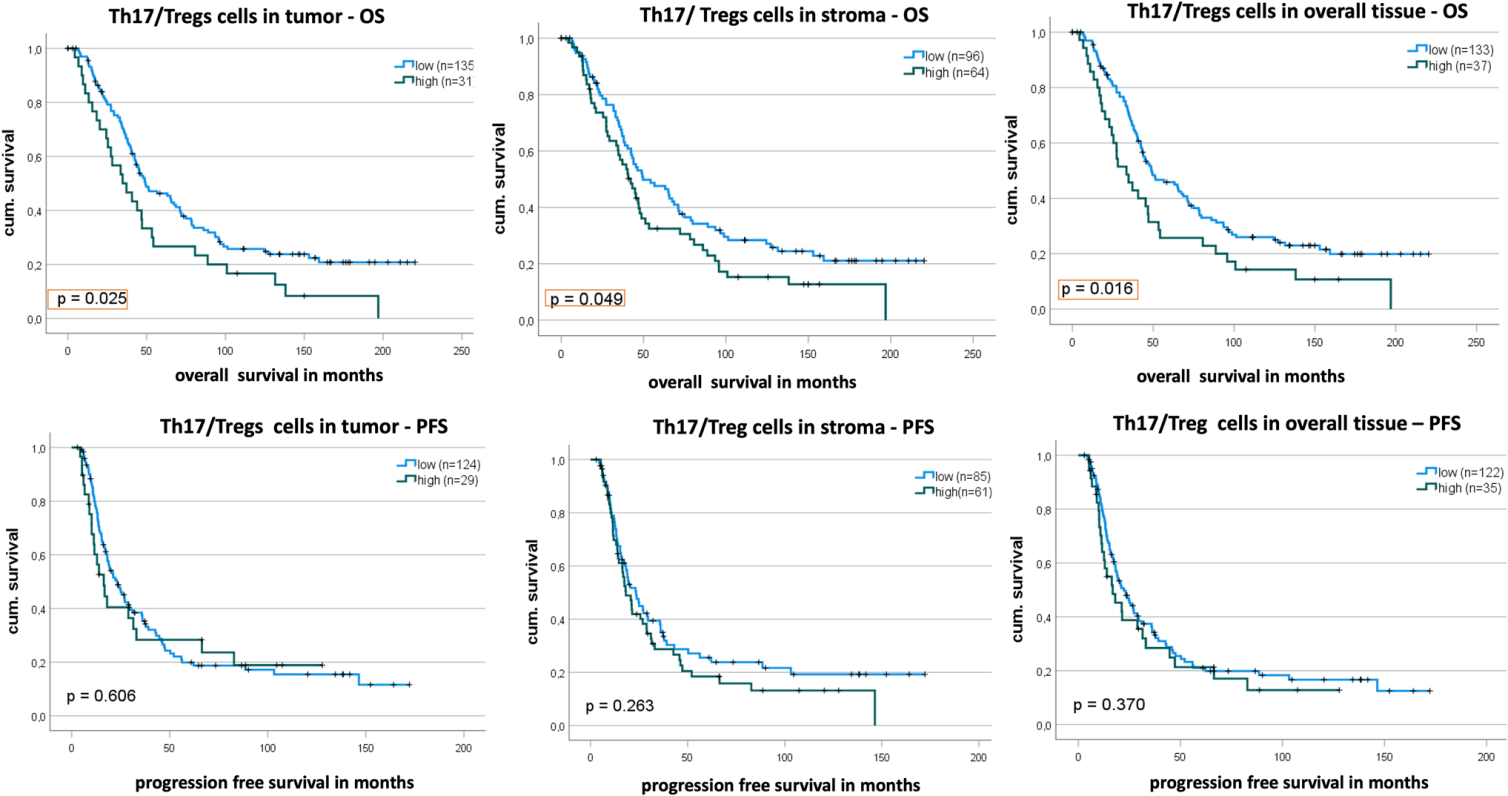
Kaplan–Meier Analysis for Th17/Tregs ratio. **A** Low ratio is significantly associated with a longer OS in tumor. **B** the ratio is significant in stroma for OS. **C** the ratio is significant in overall tissue for OS. **D**–**F** The ratio is not associated with progression free survival

We examined whether the ratio of both marker densities would yield a prognostic effect. Interestingly this turned out positive. The Th17/Treg ratio had a significant prognostic impact on the overall survival in all three Kaplan–Meier analyses (Fig. 7A, B, C). In other words, patients who had a lower ratio (higher Tregs compared to lower Th17) had an increased overall survival (*p* = 0.025, 0.049 and 0.016 for tumor, stroma and overall tissue respectively). Median OS for patients stratified to the lower ratio group was 49 months (95% CI 32.9–65.6, 95% CI 29.8–69.5, 95% CI 33.3–64.5) for tumor, stroma and overall tissue respectively. While the patients grouped above the cut-off value had a median OS of 35 months (95% CI 17.9–51.5) for tumor, 42 months (95% CI 32.7–51.7) in stroma and 33 months (95% CI 22.1–44.9) in overall tissue. After accounting for the known confounders, these relationships were not significant anymore. We also investigated whether the progression free survival can be predicted by the ratios, however, this was not the case in any of the three settings (Fig. 7D, E, F).

### In silico reproduction of prognostic significance on the mRNA level

**Fig. 8:**
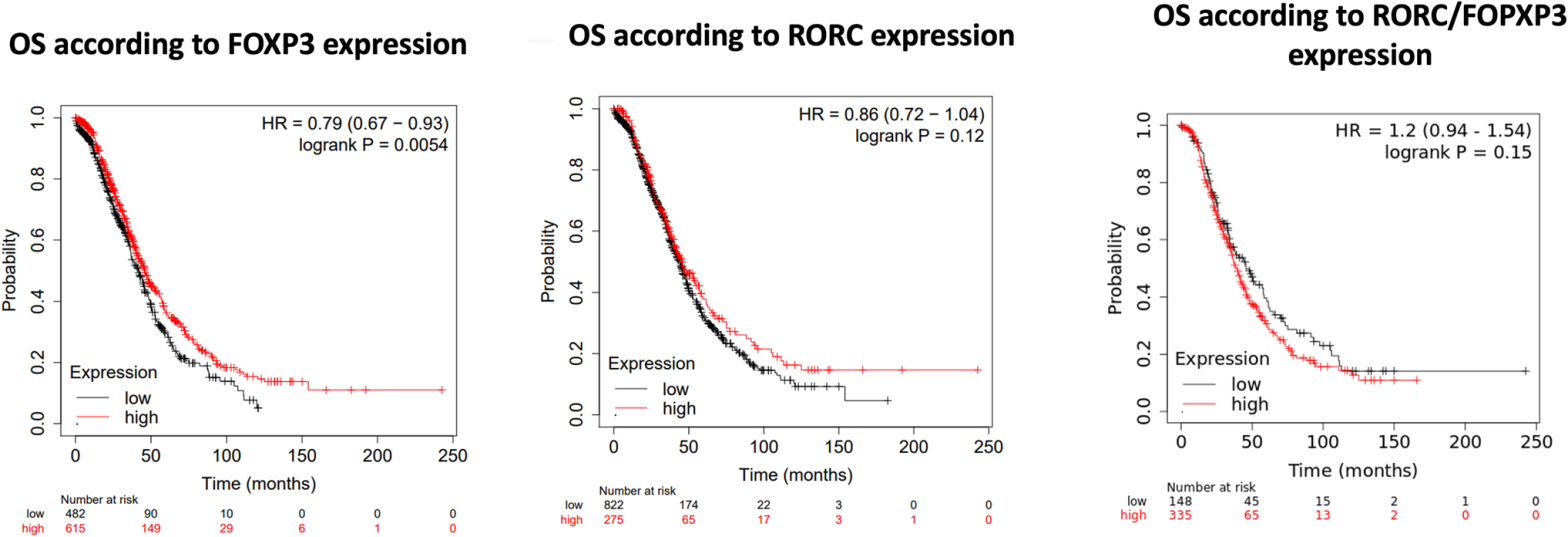
Prognostic impact of the gene expression with overall survival as outcome. **A** *FOXP3* gene (significant) **B** *RORC* gene (not significant) **C** *RORC/FOXP3* ratio (not significant)

Finally, we analyzed publicly available mRNA data to validate the observed findings from protein level on gene expression level. These gene expression patterns together with clinical data strongly support the results that were observed in Fig. 5 and Fig. 7 for overall survival. According to mRNA expression levels, patients with higher expressed FOXP3 gene had a significantly higher overall survival in comparison to those patients with lower expressed FOXP3 (*p* = 0.005) (Fig. 8A). For patients with lower expressed RORC gene the results do not show any statistical significance for a longer OS (*p* = 0.12). However, from Fig. 8, we observe the trend that higher expression of RORC tends to increase the survival rate. For the ratio of RORC:FOXP3 the gene levels are not significantly associated with a prolonged overall survival (*p* = 0.15) (Fig. 8C). When restricting the analysis to patients from FIGO stage III/IV, we observed a significant impact on the OS of patients based on FOXP3 and RORC expression, however not for their ratio (see S5).

## Discussion

In this study, we systematically analyzed the impact of the potential prognostic biomarkers Th17 and Tregs in high-grade serous ovarian carcinoma. In brief, our findings demonstrate that higher levels of Treg cells are associated with a significantly increased overall survival of patients with HGSC. This was observed in intratumoral as well as in stromal areas of the TMA cores and was additionally supported by in silico validation for mRNA using the Kaplan–Meier plotter. For Th17; we did not observe statistically significant impact neither on the protein levels, nor in in silico validation on mRNA levels. Our results demonstrate that Tregs serve as robust favorable prognostic biomarkers for HGSC.

To our knowledge, we are the first to examine the prognostic influence of specific TILs subsets in ovarian carcinoma using computational methods and differentiating between tumor and stroma tissue in the analyses. Our results on the favorable prognostic value of Tregs are in line with previous studies (Leffers et al. 2009; Milne et al. 2009). Milne et al. have concluded from their IHC study in 2009 that intratumoral FOXP3 + T cells are associated with disease specific survival, yet the study cohort lacked FIGO IV stage patients. Moreover, they only used FOXP3 as a marker to detect Tregs. However, today it is known that other subsets of TILs express FOXP3 as well. Our study overcomes both limitations: by using the CD25/FOXP3 double immunohistochemistry, we were able to target Tregs specifically and the inclusion of FIGO stage IV patients in the survival analysis allowed us to generalize the prognostic value of Tregs over all FIGO stages. Moreover, Leffers et al. reported an association with prolonged disease-free survival with higher levels of Tregs, again using FOXP3 as the only marker. Additionally, their study cohort was composed of 117 patients that had ovarian carcinoma of various histology types (Leffers et al. 2009), which exhibit distinct clinical properties that are potential confounders. Our study strengthens and refines the existent findings, as we used a larger cohort size and focused exclusively on high-grade serous histology type of ovarian cancer. Other authors did not observe a protective role of Tregs in HGSC (Adams et al. 2009; Knutson et al. 2015), neither in the peripheral blood, nor in tumoral tissue itself (Winkler et al. 2015). In contrast, there is existing evidence on the adverse effect of intraepithelial Tregs on patient survival, such as demonstrated by Sato et al; however, the difference was not found to be significant (Sato et al. 2005). Possible reasons for the differences between their results and our observations might be due to methodologic differences of the studies, such as the usage of snap-frozen specimens and a less precise cut-off point for patient stratification. Consistent with previous studies (Leffers et al. 2009; Fialová et al. 2013), we observed significantly higher levels of Tregs in samples that were classified as advanced FIGO stages. This phenomenon was as well reported in other types of female reproductive organ cancer such as cervical cancer (Vattai et al. 2022). This observation suggests that during tumor progression, Tregs accumulate in the tissue. Most cases of ovarian cancer are not diagnosed before FIGO III or FIGO IV, and since we have found a particular significance for these stages which is in line with the clinical problem of late-diagnosed ovarian carcinoma, Tregs could represent a starting point for new therapeutic approaches.

The prognostic value of Th17 has already been studied in various cancer entities, where its expression was shown to be associated with a better prognosis (Knutson et al. 2015; Winkler et al. 2015). Surprisingly, our findings did not confirm this prognostic value of Th17 cells in HGSC. Consistent with our results, Bilska et al. found that the clinical relevance of Th17 cells in ovarian cancer and their association with advanced tumor stages was insignificant (Bilska et al. 2020). Moreover, our results are supported by the data from a study conducted by Winkler et al. who concluded that an increased percentage of Th17 cells in ovarian tissue does not influence the time of survival of patients with ovarian cancer. We refine their findings, as their study cohort included only 60 women with mixed histology types of epithelian ovarian cancer (Winkler et al. 2017).

Additionally, we observed a weak inverse correlation between Th17 and Tregs, indicating that these two subtypes of TILs are interrelated to one another. In line with this discovery, Kryczek et al. found a strong inverse association between the two cell types in ovarian cancer (Kryczek et al. 2009). Interestingly, some of their other results contradict our findings: they concluded that IL-17 predicts an improved patient survival, a finding our data does not yield.

Moreover, we demonstrated that Th17/Treg ratio was associated with improved patient survival, which adds to the results from Sato et al., who have shown that a CD8/Treg ratio can be used to predict patient survival in ovarian cancer (Sato et al. 2005). As Th17 cells are a subpopulation of TILs, we wanted to investigate whether the effector-suppressor-ratio might be a more important indicator for the patient survival than individual cell frequencies. Nevertheless, a confounding major effect of Tregs in this ratio cannot be excluded. It is well known that the microenvironment of tumors is highly complex, and interactions are not fully understood yet, making therapeutic approaches challenging.

None of the existing studies focused on lymphocytes from stromal tissue surrounding the tumor area. During our analyses, we clearly observed that the prognostic impact depends on whether the cell level is quantified inside the stromal or the intratumorally tissue. Prior findings also revealed substantial differences in immune cell compositions in different regions of the HGSC (Corvigno et al. 2021). Hence, for future studies we would strongly recommend performing compartment separation prior to further analysis. As this might reveal differentiated functional mechanisms of lymphocytes that surround the tumor from those that are inside of it and provide novel clinically relevant marker. Specifically, the use of algorithm-based cell detection leads to a substantial increase in precision and efficiency of quantification of different immune cell populations and provides the basis for a reproducible downstream analysis compared to manual scoring. An approach like this is highly recommendable as it has already led to some interesting discoveries for various tumor entities (Farace et al. 2011).

The potential use of TILs as prognostic biomarkers for predicting the patients’ survival relies on the selection of a suitable cutoff point. Unlike previous studies of TILs in ovarian cancer, we used an optimal cut-off value and assessed the robustness of the marker by computing the number of cut-offs that yielded a significant difference between the groups. As we determined the cut-off by including the outcome variable rather than just a distribution-based cutoff, we computed separate cut-off values for PFS and OS. It must be noted that this calculation of separate cut-offs is not useful in clinical practice. Instead, a single sensible cut-off should be determined here. We are aware that dichotomization based on a cut-off that yields the largest possible survival discrepancy is associated with type II errors, to account for that we considered an area of successive significant cut-offs and selected the best one. The reported cut-offs need to be validated on further study populations.

Our study comes with some potential limitations that need to be considered. While the double CD25/FOXP3 staining led to clear identification of positive and negative cells, the outcome for the nuclear RORγt staining was less precise. And although we have observed a good agreement between the automatized cell detection and manual cell count of two experienced pathologists, even with automated cell detection methods, positive cells were hard to identify, which possibly resulted in potential bias in the downstream analysis. We suggest that further research on RORγt in a different patient cohort will reveal more clarity on this relationship. In general, this study is limited by the number of participants and additional validation on larger sample sizes will be beneficial to confirm our findings. At this point, it is important to consider that survival analyses are a purely statistical approach. Thus, even though Tregs and the Th17/Treg ratio are significant prognostic factors, it does not provide insights into the mechanisms of their functioning or the complex T cell differentiation paths. Besides, a dominant effect of Tregs in the prognostic impact of Th17/Treg ratio cannot be excluded, hence further studies will be required to understand the interplay of Tregs and Th17 in ovarian cancer.

In conclusion, our data underlines that regulatory T cells are an independent favorable prognostic biomarker in high-grade serous ovarian cancer. These results give the foundation for the development of future immunotherapies targeting high grade serous ovarian carcinoma. Furthermore, we suggest that further studies should investigate the precise biological functions and the interaction of Tregs and Th17 cells in the context of ovarian cancers.

## Supplementary Information

Below is the link to the electronic supplementary material.

Supplementary file1 (PPTX 842 KB)

## Data Availability

The datasets generated and analyzed during the current study are available from the correspondent author on reasonable request.
