# Supplementary material for: Prognostic value of regulatory T cells and T helper 17 cells in high grade serous ovarian carcinoma
Source: J Cancer Res Clin Oncol. 2022 Jun 28;149(6):2523–36. doi: 10.1007/s00432-022-04101-2 (PMC10129928; doi:10.1007/s00432-022-04101-2)
Supplement: Supplementary file 1 — Supplementary file1 (PPTX 842 KB) [file 432_2022_4101_MOESM1_ESM.pptx]

## Slide 1
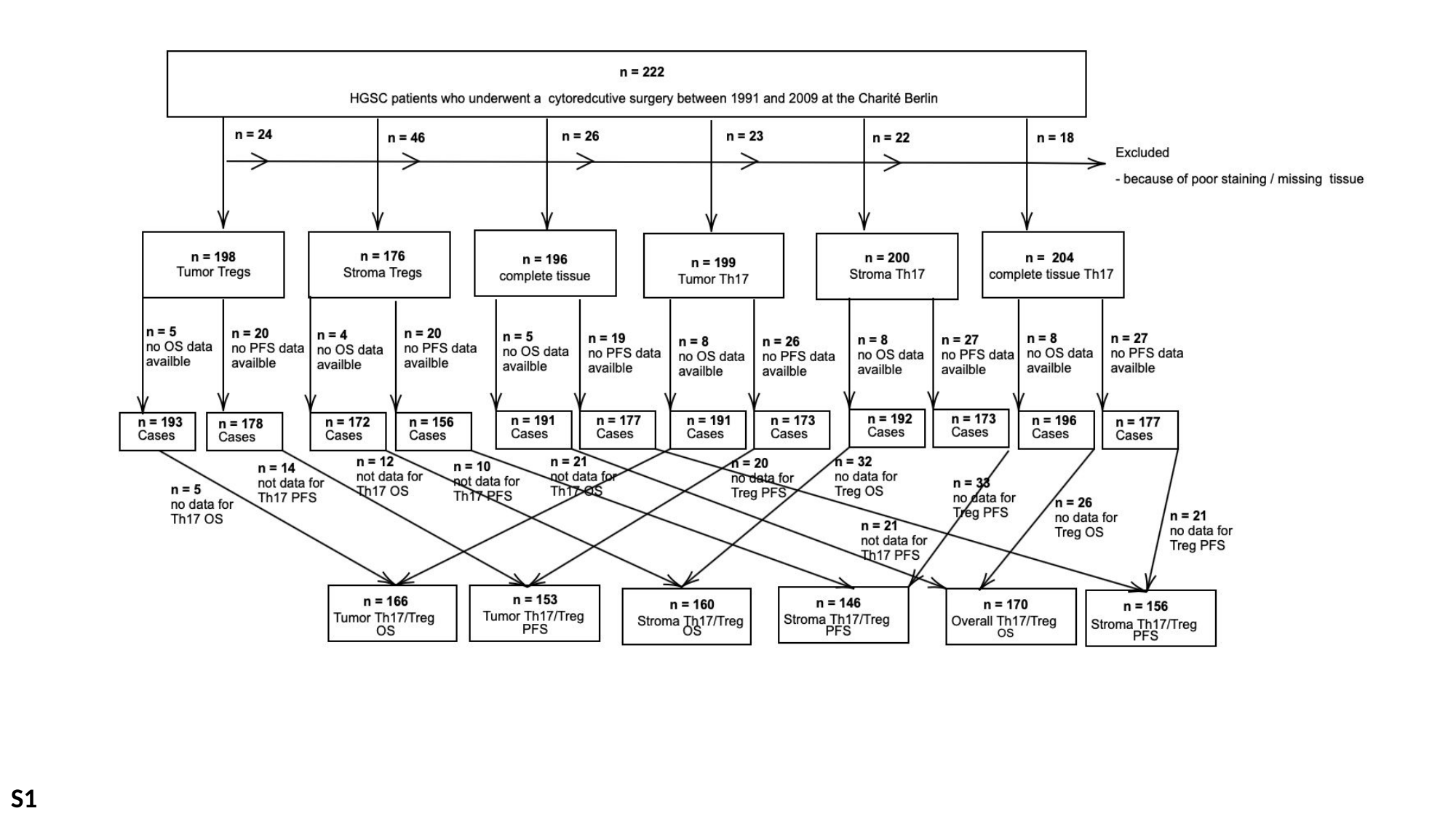

S1

## Slide 2
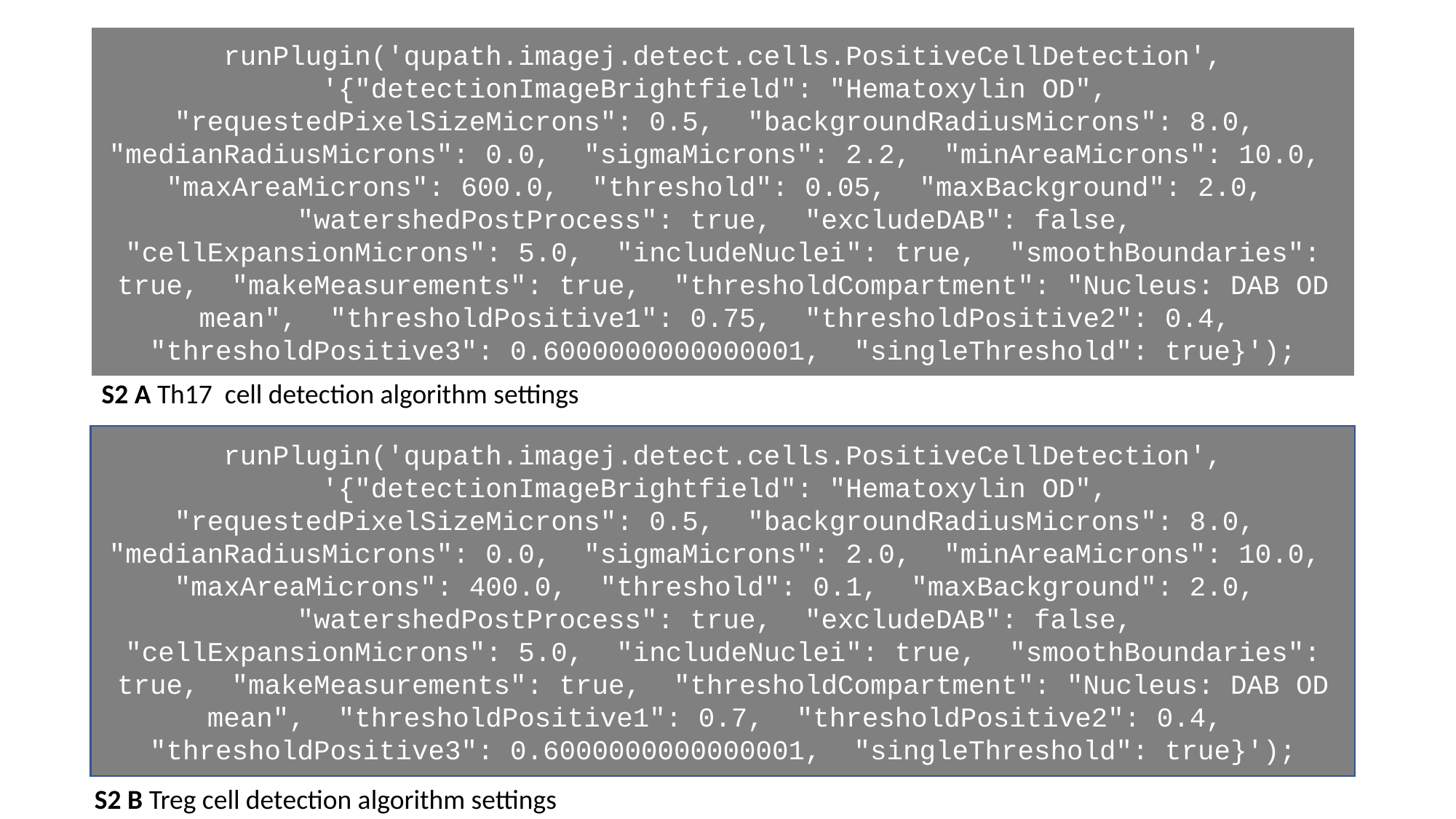

runPlugin('qupath.imagej.detect.cells.PositiveCellDetection', '{"detectionImageBrightfield": "Hematoxylin OD", "requestedPixelSizeMicrons": 0.5, "backgroundRadiusMicrons": 8.0, "medianRadiusMicrons": 0.0, "sigmaMicrons": 2.2, "minAreaMicrons": 10.0, "maxAreaMicrons": 600.0, "threshold": 0.05, "maxBackground": 2.0, "watershedPostProcess": true, "excludeDAB": false, "cellExpansionMicrons": 5.0, "includeNuclei": true, "smoothBoundaries": true, "makeMeasurements": true, "thresholdCompartment": "Nucleus: DAB OD mean", "thresholdPositive1": 0.75, "thresholdPositive2": 0.4, "thresholdPositive3": 0.6000000000000001, "singleThreshold": true}');
#
S2 A Th17  cell detection algorithm settings
runPlugin('qupath.imagej.detect.cells.PositiveCellDetection', '{"detectionImageBrightfield": "Hematoxylin OD", "requestedPixelSizeMicrons": 0.5, "backgroundRadiusMicrons": 8.0, "medianRadiusMicrons": 0.0, "sigmaMicrons": 2.0, "minAreaMicrons": 10.0, "maxAreaMicrons": 400.0, "threshold": 0.1, "maxBackground": 2.0, "watershedPostProcess": true, "excludeDAB": false, "cellExpansionMicrons": 5.0, "includeNuclei": true, "smoothBoundaries": true, "makeMeasurements": true, "thresholdCompartment": "Nucleus: DAB OD mean", "thresholdPositive1": 0.7, "thresholdPositive2": 0.4, "thresholdPositive3": 0.6000000000000001, "singleThreshold": true}');
S2 B Treg cell detection algorithm settings

## Slide 3
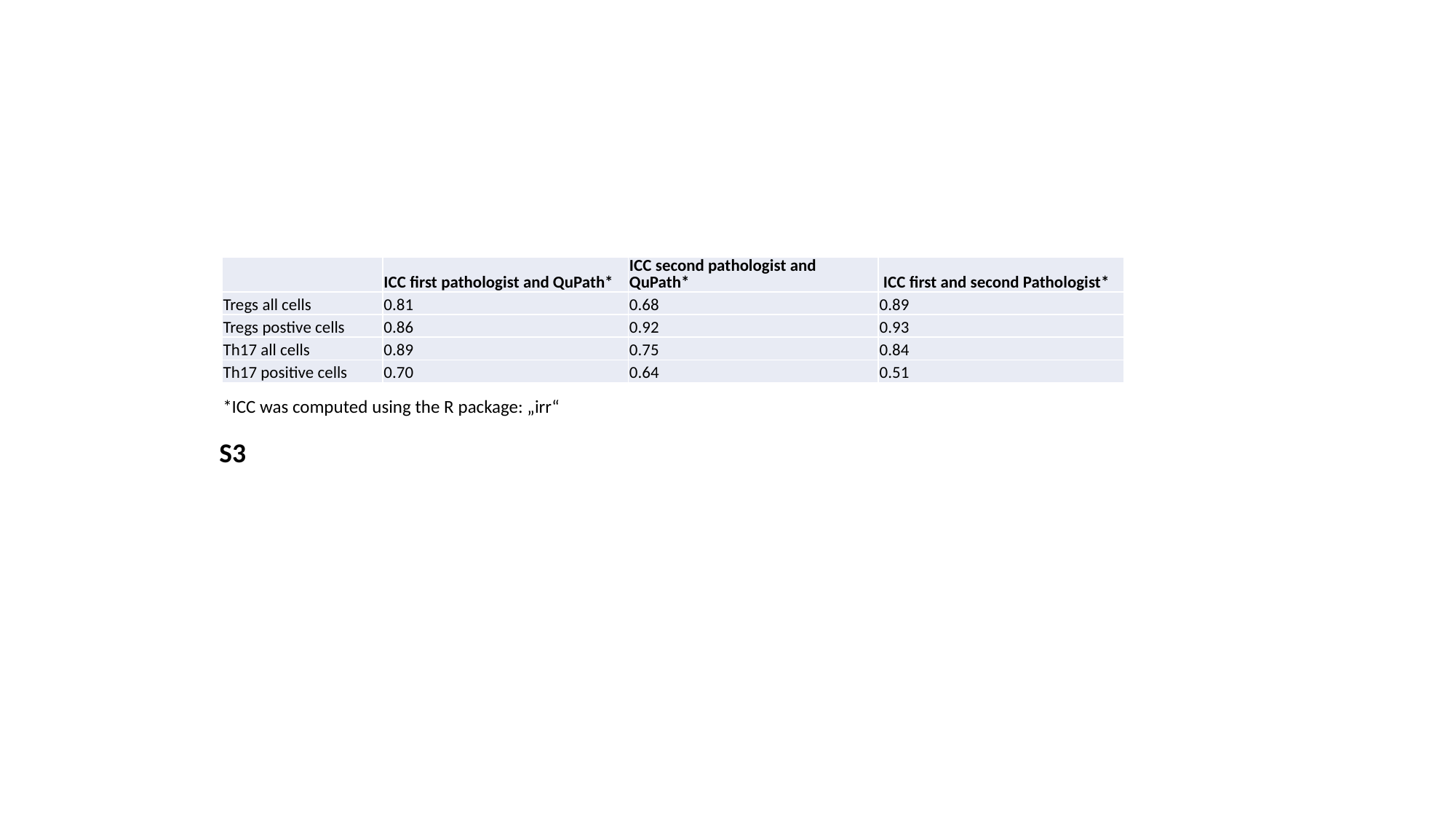

| | ICC first pathologist and QuPath\* | ICC second pathologist and QuPath\* | ICC first and second Pathologist\* |
| --- | --- | --- | --- |
| Tregs all cells | 0.81 | 0.68 | 0.89 |
| Tregs postive cells | 0.86 | 0.92 | 0.93 |
| Th17 all cells | 0.89 | 0.75 | 0.84 |
| Th17 positive cells | 0.70 | 0.64 | 0.51 |
*ICC was computed using the R package: „irr“
S3

## Slide 4
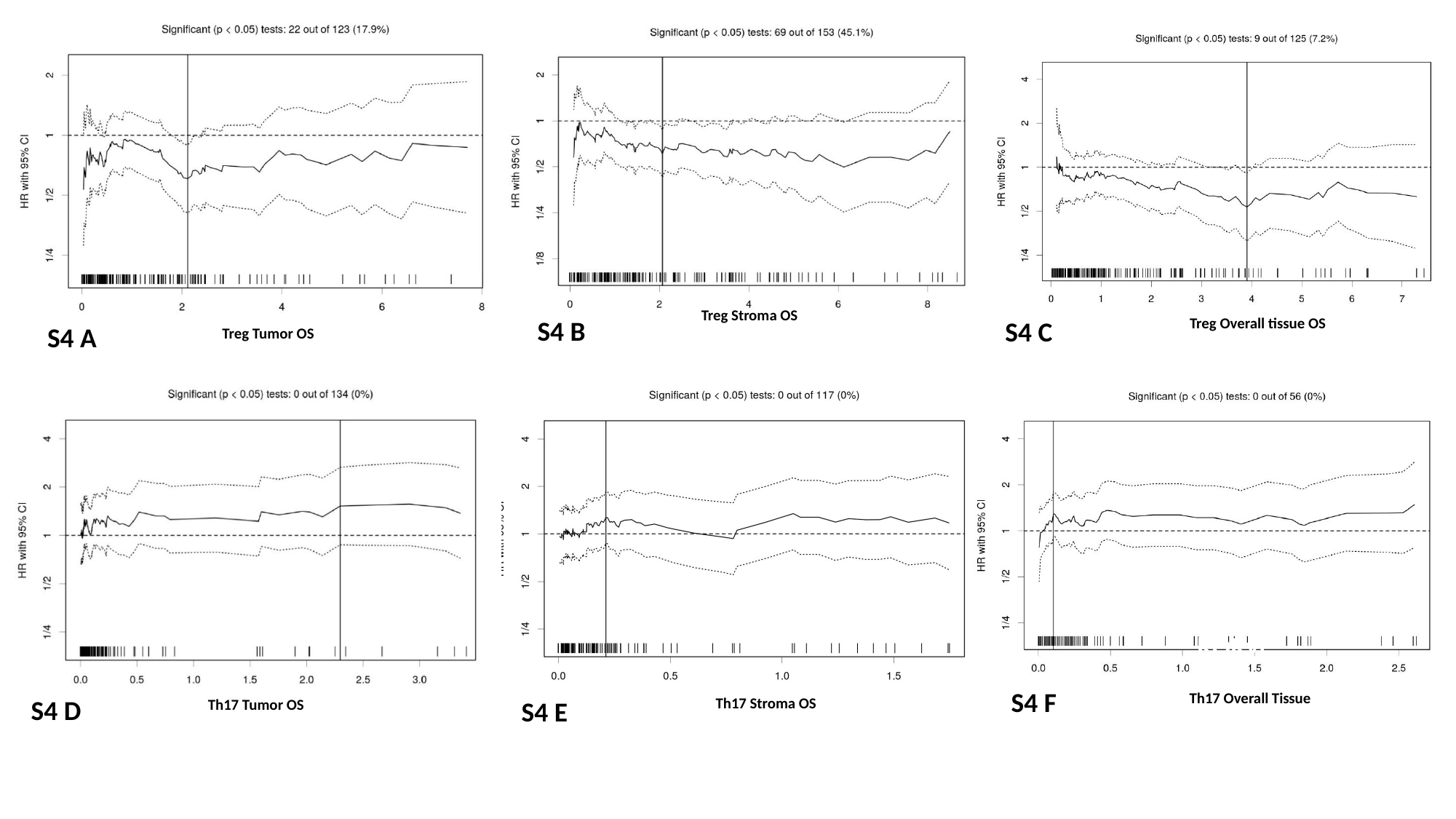

S4 B
Treg Stroma OS
Treg Overall tissue OS
S4 C
S4 A
Treg Tumor OS
S4 F
Th17 Overall Tissue
RORγt+ Stroma
S4 D
Th17 Stroma OS
S4 E
Th17 Tumor OS

## Slide 5
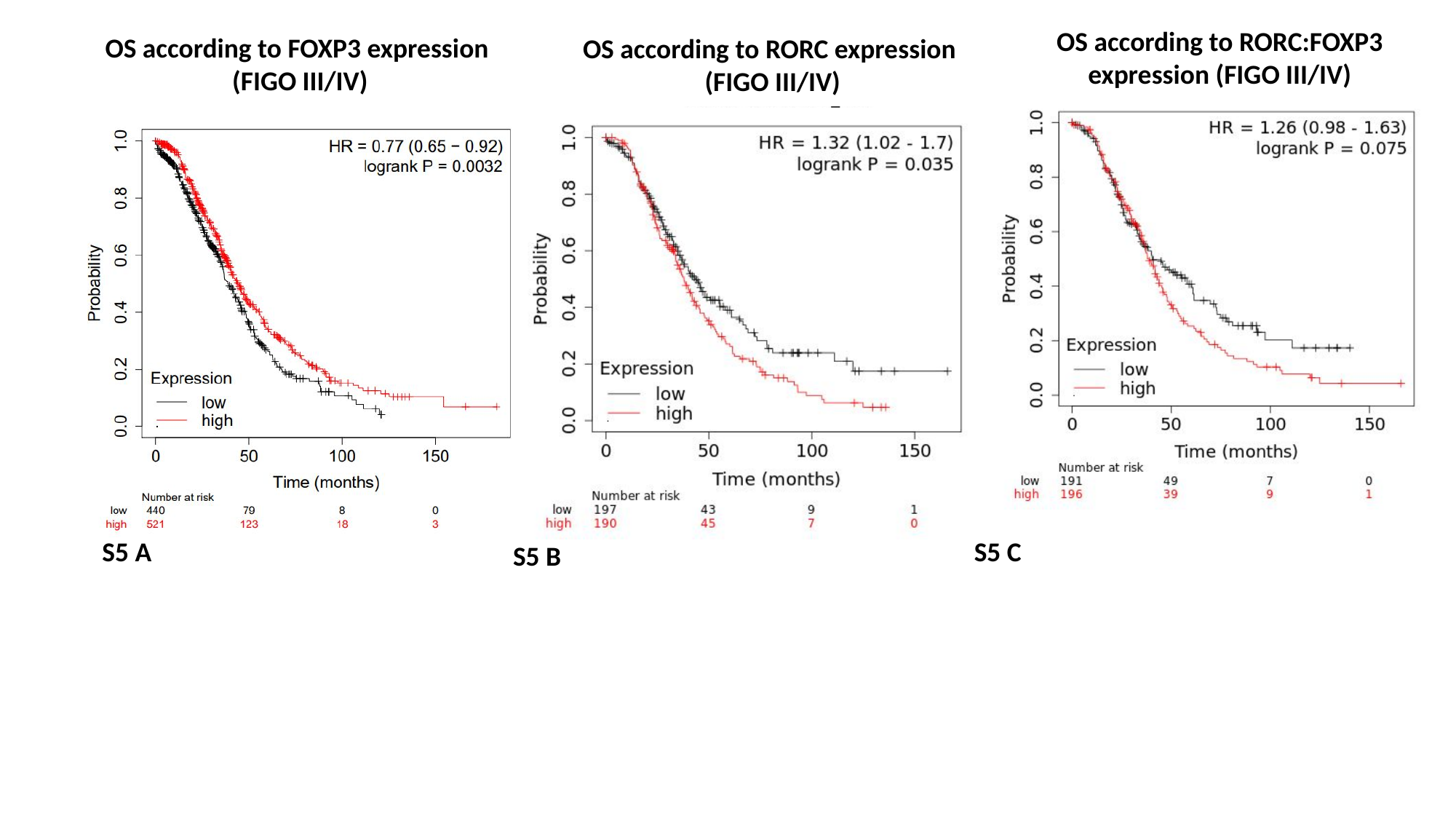

OS according to FOXP3 expression
 (FIGO III/IV)
OS according to RORC:FOXP3 expression (FIGO III/IV)
OS according to RORC expression
 (FIGO III/IV)
OS according to RORC:FOXP3 ratio
OS according to RORC expression
S5 A
S5 C
S5 B
